# Evaluating the Lived Experiences of Older Adults with Fall-Monitoring Technology

**DOI:** 10.1093/geroni/igaf122.3757

**Published:** 2025-12-31

**Authors:** Alisha Johnson, Chang-Chun Chen, K Melinda Fauss, Wung Shu-Fen

**Affiliations:** University of Missouri, Columbia, Missouri, United States; University of Arizona, Tucson, Arizona, United States; Sinclair School of Nursing, Columbia, Missouri, United States; University of California, Davis, Betty Irene Moore School of Nursing, Sacramento, California, United States

## Abstract

Every year in the US, 30% of older adults experience falls, resulting in three million visits to emergency departments. Fall-monitoring technologies have proven effective in improving the quality of life for older adults, allowing them to age in place. Though these technologies enhance safety and keep caregivers informed, they also raise concerns regarding autonomy and privacy. However, there is limited understanding of the perspectives of older adults living with these technologies. We used an interpretive phenomenological approach, interviewing eight older adults, with and without dementia, residing in two independent living facilities who had experienced fall-detection technologies for the previous 9-12 months. The interviews focused on understanding how technology influences their self-identity, ability to live independently, safety, and age-in-place. To ensure trustworthiness, field-notations, reflexive journaling, and participant check-backs were used. Several themes emerged reflecting the influence of technologies on lived experiences: the freedom to age in place, the necessity for active intervention, individual approaches to technology based on personality/cognition, and feelings of discomfort and fear. Older adults were more inclined to engage with monitoring technologies if they were open to intervention, had their concerns addressed, and perceived the technology as useful for enabling aging-in-place. Older adults, with and without dementia, are often the target audience for monitoring technologies, yet their perspectives are frequently overlooked. This study significantly enhances the understanding of effective implementation strategies for monitoring technologies. It serves as a valuable resource for individuals and facilities seeking successful integration of fall-detection and prevention technologies to support older adults to age-in-place.

